# Early onset sebaceous carcinoma

**DOI:** 10.1186/1746-1596-6-81

**Published:** 2011-09-05

**Authors:** Dongjin Sung, Sara A Kaltreider, Federico Gonzalez-Fernandez

**Affiliations:** 1 Department of Ophthalmology, Ross Eye Institute and State University of New York, 1176 Main Street, Buffalo, NY, 14209, USA; 2 Medical Research Service, Veterans Affairs Medical Center, 3495 Bailey Ave., Buffalo, NY, 14215, USA; 3 Department of Ophthalmology, University of Virginia Health Sciences Center, 1215 Lee Street, Charlottesville, VA, 22908, USA; 4 Department of Pathology & Anatomic Sciences, 3435 Main Street, State University of New York, Buffalo, NY, 14214, USA

## Abstract

**Background:**

Ocular sebaceous carcinoma can masquerade as benign lesions resulting in delay of diagnosis. Early recognition is even more difficult in young patients where the disease rarely occurs. Here, we provide a clinicopathological correlation of ocular sebaceous carcinoma in a young individual lacking history of hereditary cancer or immunosuppression.

**Findings:**

A detailed histopathological study including *p53 *DNA sequencing was performed on an aggressive sebaceous carcinoma presenting in a healthy 32 year-old Caucasian woman. She had no history of retinoblastoma, evidence for a hereditary cancer syndrome, or radiation therapy. However, she potentially was at risk for excessive UV light exposure. A detailed review of the literature is also provided.

A moderately well differentiated sebaceous carcinoma was established histopathologically arising from the meibomian gland of the upper eyelid. In most areas, the cytoplasm contained small but distinct Oil-red-O positive vacuoles. Direct sequencing of *p53 *identified a G:C→A:T mutation at a dipyrimidine site. The mutation results in substitution of arginine for the highly conserved glycine at residue 199 located at the p53 dimer-dimer interface. Energy minimization structural modeling predicts that G199R will neutralize negative charges contributed by nearby inter- and intramonomeric glutamate residues.

**Discussion:**

This study points to the importance of recognizing that sebaceous carcinoma can occur in young patients with no evidence for hereditary cancer risk or radiation therapy. The G199R substitution is anticipated to alter the stability of the p53 tetrameric complex. The role of UV light in the etiology of sebaceous carcinoma deserves further study. Our findings, taken together with those of others, suggest that different environmental factors could lead to the development of sebaceous carcinoma in different patients.

## Background

Sebaceous carcinoma is generally considered to be a tumor of older patients (mean age, 73 years of age) [1]. In the ocular adnexa, this highly malignant neoplasm often masquerades as common benign lesions delaying appropriate treatment [2-4]. For unknown reasons, sebaceous carcinoma is more prevalent in the ocular adnexa than elsewhere in the body. It can arise from the meibomian glands, glands of Zeis, caruncle, skin of eyelid and eyebrow, lacrimal gland, or conjunctiva [4-8]. Although its etiology is largely unknown, it has been associated with Asians [4,9], Muir-Torre syndrome [10-14], *Rb *and *p53 *mutations [3,15,16], HIV [17], and HPV [3,18]. The apparent increased rate of sebaceous carcinoma in Asians/Pacific Islanders is being challenged by recent studies indicating that the tumor is more common in Whites [1,9], and showing a distribution consistent with sunlight exposure [19].

Early recognition of sebaceous carcinoma is often challenging, requiring close collaboration between ophthalmologist and pathologist. Early stages of the disease can consist of only Pagetoid extension without tumefaction [3]. This *in situ *stage can be mistaken for blepharoconjunctivitis. Sebaceous carcinoma is often clinically misdiagnosed as chalazion, a common lipogranulomatous lesion of the eyelid. Furthermore, it is important to recognize that Merkel and basal cell carcinomas can mimic sebaceous carcinoma microscopically. Therefore, early diagnosis of sebaceous carcinoma requires appreciation of its varied clinical presentation, and recognition of entities that can mimic it histopathologically [4].

Here we describe a sebaceous carcinoma presenting in a 32 year-old woman. This case was previously included in a series of seven cases [3]. Here, we revisit this case to provide further clinical, histopathological and molecular studies. The case emphasizes that this highly aggressive cancer can occur in young patients without history of retinoblastoma, radiation therapy, or hereditary cancer syndrome. Finally, the case suggests that the role of UV radiation and *p53 *mutations in sebaceous carcinoma deserve further study.

## Findings

### Clinical history

A healthy 32 year-old Caucasian woman was referred for a recurrent chalazion initially diagnosed 3 months prior. She had no previous ocular or medical problems. There was no significant family history of cancer (a maternal uncle had hepatocellular carcinoma; her paternal great grandmother reportedly had stomach cancer). For 14 years she was a cosmetologist, and for 9 of those years regularly used a home UV tanning bed.

**Figure 1:**
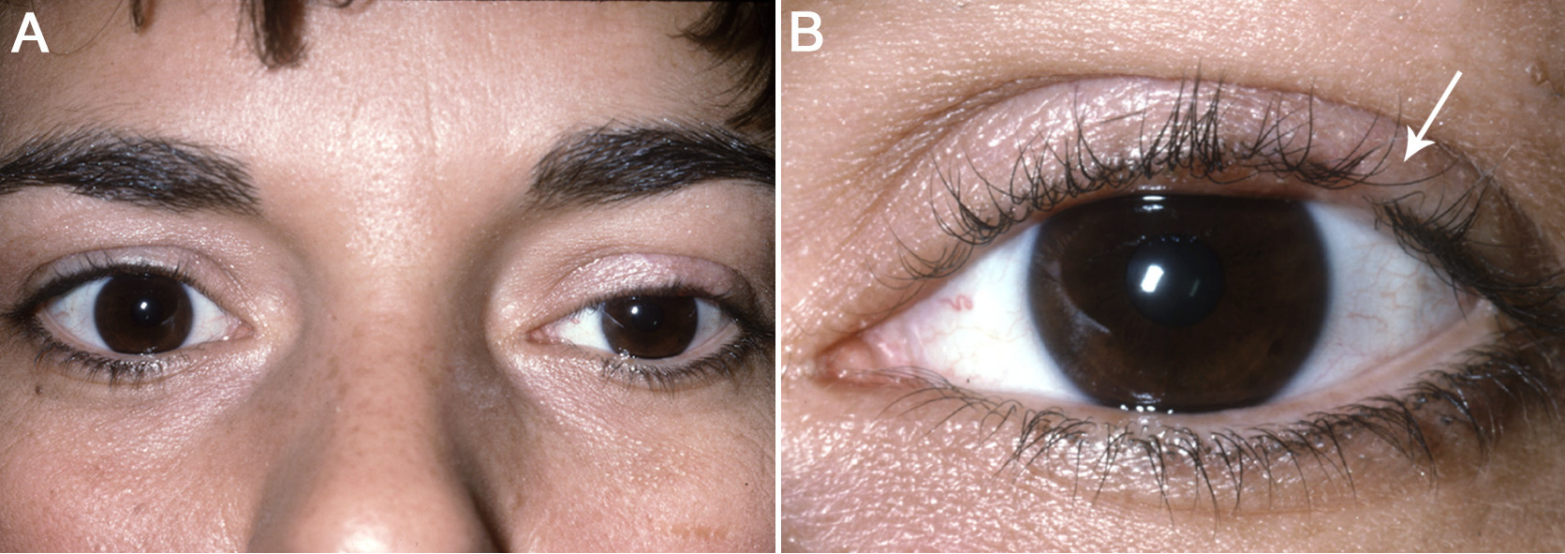
**32 year-old cosmetologist presented with 3 month history of treatment for presumed chalazion**. **A) **The left upper eyelid showed 2 mm of ptosis with swelling. **B) **Focal loss of eye lashes was appreciated (arrow).

Physical examination showed 2.0 mm of left eyelid ptosis, and thickening of the tarsus and tarsal conjunctiva (Figure 1A). Focal loss of eyelashes on the affected eye was also appreciated (arrow in Figure 1B). The remainder of her examination was unremarkable. She had no bulbar injection or corneal findings. There was no ocular discomfort, loss of vision, or motility deficit. Biopsy of the left upper eyelid demonstrated sebaceous carcinoma (see below). Resection with frozen section control was performed. The resection included the entire upper lid with the canalicular system. All eyelid tissue from the medial to lateral canthal angle, and up to the level of the superior fornix was excised. Four months later, she presented with left eye proptosis and pain. Her care was complicated by orbital extension requiring exenteration of the left socket with neck dissection. The tumor had extensively infiltrated the orbital fat, and extraocular muscles. The parotid gland and three of seven anterior neck lymph nodes were also involved. Despite this surgery, and adjuvant radiation treatment, she died 2.5 years later of her disease.

### Pathologic and molecular observations

**Figure 2:**
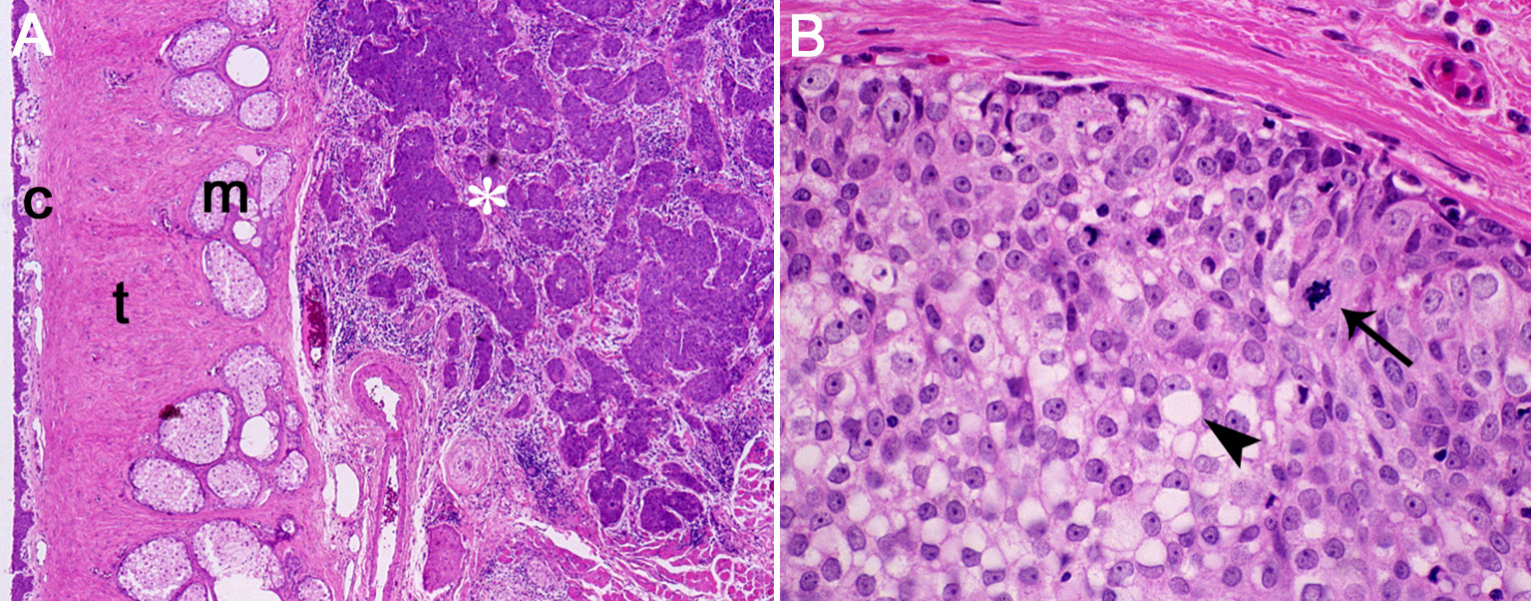
**Hematoxylin and eosin stained section from a full-thickness wedge resection**. **A) **The tumor, which had no connection with the conjunctival epithelium or epidermis, consisted of numerous lobules of invasive disease throughout the eyelid. C, conjunctiva; t, tarsus; m, meibomian gland; asterisk, tumor lobules. **B) **High magnification shows a moderately differentiated sebaceous carcinoma with cytoplasmic lipid vacuoles (arrowhead), and abnormal mitotic figures (arrow).

**Figure 3:**
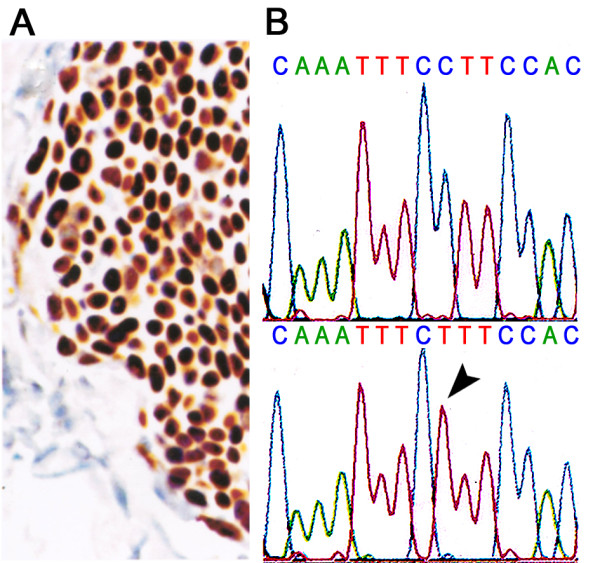
**Analysis of p53 from the patient's tumor**. **A) **Elevated nuclear p53 expression in tumor cells is shown in this paraffin section by avidin-biotin complex immunohistochemistry. Control sections, which received non-immune serum, showed no immunoreactivity (data not illustrated). **B) **Automated fluorescent DNA sequencing analysis (antisense strand) demonstrated a missense mutation in exon 6 of *p53*. The upper and lower panels show the wild-type and tumor sequences respectively. The arrowhead shows the C→T mutation in the antisense strand.

The initial full thickness wedge biopsy revealed a moderately well differentiated sebaceous carcinoma (grade II [20]). Subsequently, the upper eyelid was removed under frozen section control between the medial and lateral canthal angles, and to the fornix superiorly. Histologically, the tumor filled the eyelid replacing most of the tarsus. The deep surgical margin was found to be focally involved. The tumor had a lobular pattern and extensively invaded the tarsus, dermis and muscle (Figure 2A). There was no Pagetoid involvement of the conjunctiva or epidermis. Mitotic figures were numerous, and often atypical in appearance (Figure 2B, arrow). In most areas, the cytoplasm contained small but distinct Oil-red-O positive vacuoles. These vacuoles did not stain with mucicarmine or periodic acid-Schiff (data not illustrated). The tumor was previously shown to stain focally for epithelial membrane antigen and p21^WAF1/CIP1^; Bcl-2 was negative as was HPV by *in situ *hybridization and PCR [3]; p53 showed striking nuclear positivity (see patient #6, table 1 of reference [3]) (Figure 3A).

**Figure 4:**
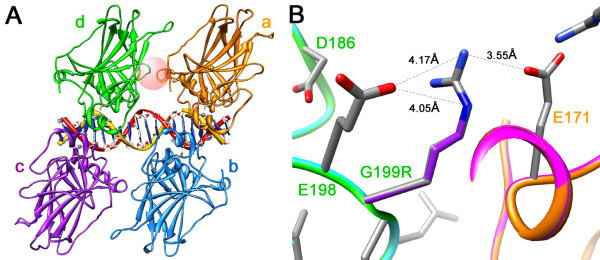
**Structural modeling shown here predicts that G199R will alter the ionic environment at the p53 dimer-dimer interface**. The X-ray coordinates for wild-type p53 were obtained from the RCSB PDB Protein Data Bank (ID 3KMD, deposited by Chen et al. (2010) [36]). The structures were produced using the UCSF Chimera package. **A) **The ribbon representation shows the quaternary structure of the p53 tetramer with bound DNA. The four p53 monomers are colored in gold (a), light blue (b), purple (c) and green (d). The DNA helix is in the center of the tetramer. P53 monomers form inter-protein contacts across the DNA axis (dimers of monomer pairs d-c and a-b), and along the DNA axis (between monomers d and a, between monomers c and b). The later are known as the "dimer-dimer" contacts. One of these interfaces is represented by a sphere between monomers d and a. G199R is located at the center of this sphere. **B**) This panel shows an *in silico *energy minimization performed within a 10Å sphere surrounding G199R. Molecular Operating Environment (MOE) was used for this analysis (release 2005.08, Chemical Computing Group, Montreal, Quebec). Energy-minimized monomers d and a are shown in magenta and cyan, respectively. The modeling predicts that the substitution of arginine for glycine at position 199 will neutralize negative charges contributed by nearby inter- and intramonomeric glutamate residues (E171 and E198, respectively).

To define the nature of the mutation responsible for the p53 nuclear accumulation, we sequenced exons 5 through 9. To do this, DNA from a tumor metastasis was extracted. Hematoxylin & eosin stained frozen sections were used to establish that the portion of the tissue used for the DNA extraction consisted only of tumor without contaminating normal tissue. The DNA was extracted and purified by acid-phenol chloroform [21]. From the purified tumor genomic DNA, *p53 *exons 5 and 6, and 7 through 9 were amplified separately by PCR, and subjected to automated fluorescence DNA sequencing using magnetic beads, and dye-primer and dye-terminating chemistries. The strategy and primers used have been previously described [3]. DNA sequencing identified a G:C→A:T (GGA to AGA) missense mutation at a dipyrimidine site in exon 6 (Figure 3B). The mutation results in substitution of arginine for the highly conserved glycine at codon 199 located at the dimer-dimer interface. Energy minimization modeling shown in Figure 4 anticipates that this substitution will significantly alter the ionic environment at the p53 dimer-dimer interface.

## Discussion

Clinically, sebaceous carcinoma has a propensity to masquerade as a variety of common benign lesions. This has contributed to an extended interval between presentation and appropriate treatment. In recent years, this gap has been closing with improved awareness of its varied clinical manifestations, and histopathological features. The present case underlines that young age should not exclude consideration of sebaceous carcinoma.

When ocular sebaceous carcinoma occurs at a young age, it is generally associated with a predisposing factor (Table 1). The youngest cases are from children with bilateral retinoblastoma (8 and 12 years). Older patients (17 and 28 years) had unilateral retinoblastoma. Although radiation therapy may have played a role, it may not be necessary as sebaceous carcinoma has occurred outside the radiation field, and in a patient who never received radiation or chemotherapy [22]. Muir-Torre syndrome, which is due to inactivation of *MSH2 *or *MLH1*, predisposes to sebaceous tumors and other neoplasms [23]. Genomic instability may provide a mechanism for the occurrence of sebaceous carcinoma in a 31 year-old man with Muir-Torre syndrome [24]. Early onset disease in the setting of HIV and steroid treatment suggests a role for immunosuppression [17,25]. In the present case, there was no history of retinoblastoma, radiation therapy or immunosuppression. Furthermore, there was no clinical or family history suggesting Muir-Torre syndrome, or another hereditary cancer syndrome. Potential risk factors for this patient were her 14-year employment as a cosmetologist, and regular home-tanning bed use for 9 of those years. Although this is speculative, a cancer risk has been associated with hair dye exposure [26], and tanning bed use [27].

**Table 1:** Risk factors of early onset (age < 40 years) ocular sebaceous carcinoma

Number of Patients	Age Range in years	Race (Number of patients)	Risk factors	References
11	8-30	C(4), U(7)	Bilat-Rb; RT	[16,20,40-46]

2	17, 28	U(2)	Unilat-Rb; RT	[47]

1	32	A(1)	Unilat-Rb	[48]

2	27, 37	U(2)	RT	[29,49]

1	31	U(1)	Muir-Torre Syn.	[12]

2	34, 36	U(2)	HIV	[17]

1	33	C(1)	Steroid*	[5]

1	20	A(1)	None	[50]

5	15-36	C(3), A(1), U(1)	U	[51,52]

1	32	C	UV, Cosmetologist	Present case

A, Asian; C, Caucasian; CT, chemotherapy; HIV, human immunodeficiency virus; Rb, retinoblastoma; RT, radiotherapy; U, unknown; *Steroid for idiopathic thrombocytopenic purpura.

Epidemiological studies suggest a role of sunlight and radiation in sebaceous carcinoma. A recent retrospective study of 1,349 cases, showed that Caucasians are more frequently affected with sebaceous carcinoma compared to Asian/Pacific Islanders and Blacks [1]. Furthermore, Rao et al. (1982) found only four Blacks among 75 patients with sebaceous carcinoma [28], and Zürcher et al. (1998) noted no black patients in their series of 43 cases (42, Caucasian; one, Chinese) [8]. Finally, sebaceous carcinoma has occurred at nonocular sites in the setting of radiation exposure [29,30] and sunlight [19]. Interestingly, the G199R substitution detected in our patient was due to a G:C→A:T mutation at a dipyrimidine site (GGA to AGA, sense strand; TCC to TCT, antisense strand). UV light is known to cause C→T substitutions at dipyrimidines, or CC→TT substitutions in the *p53 *gene [31]. Although this class of mutation is typical of UV light induced mutations, it does not prove that the mutation was caused by UV exposure. Among 11 cases of sebaceous carcinoma with documented *p53 *mutations, 5 showed C→T mutations at dipyrimidine sites [15]. An additional reported case from a 75 year-old woman showed a G:C→T:A typical of bulky carcinogens [3]. The diversity of mutations suggests that different mechanisms may play a role in different cases (Table 2).

**Table 2:** Known p53 mutations in ocular sebaceous carcinoma

Age/Sex	Site	Mutation			
		
		Location	Codon	Amino acid	Type
83 yrs/F [15]	LL	Exon 5	TACt to TAGt	Y126stop	C:G→G:C

75 yrs/M [15]	ul	Exon 5	GTG to ATG	V173M	G:C→A:T

36 yrs/F [15]	LL	Exon 5	CGC to CAC	R175H	G: C→A:T

78 yrs/M [15]	ul	Exon 6	CAT to CGT, AGT to GGT	H193R, S215G	A:T→G:C A:T→G:C

32 yrs/F*	ul	Exon 6	GGA to AGA	G199R	G:C→A:T

85 yrs/M [15]	LL	Exon 7	GGC to AGC	G245S	G:C→A:T

42 yrs/M [53]	NP	Exon 7	cCGG to cTGG	R248Y	C:G→T:A

76 yrs/F [15]	LL	Exon 7	cCGG to cTGG	R248W	C:G→T:A

61 yrs/M [15]	LL	Exon 8	gGTG to gATG	V272M	G:C→A:T

61 yrs/F [15]	ul	Exon 8	TGT to TAT	C275Y	G:C→A:T

75 yrs/F [3]	LL	Exon 8	TGT to TTT	C277F	G:C→T:A

47 yrs/F [15]	ul	Int 1	TGgt to TGtt	NA	G:C→T:A

68 yrs/M [15]	LL	Int 1	agGT to aaGT	NA	G:C→A:T

ul, Upper eyelid; LL, Lower eyelid; NA, Nonapplicable; NP, Not provided;

Underline, dipyrimidine; * present case

Clinical data and molecular studies suggest that G199R likely interferes with p53 function. Early X-ray structures of p53 showed that the known "hot-spot" mutations are often involved in DNA binding. However, codon 199 lies outside of the direct DNA-protein binding site. Nevertheless, missense mutations of this codon are listed in 48 tumors in the International Agency for Research on Cancer database [32]. Of those, 12 are G199R substitutions [32]. In many of these cases, a link between G199R and p53 function can be made. Cyclophosphamide induced bladder cancer is more commonly associated with G199R compared to sporadic, smoking-related, and schistosomiasis-linked tumors [33]. In a study of giant cell glioblastoma multiforme, G199R was more often associated with evidence for microsatellite instability compared to other mutations [34]. G199R, which was observed in BRCA-1 associated familial breast cancer [35], shows reduced activity in yeast transactivation capacity assays. Thus, although not directly involved in DNA-protein binding, G199 appears to have a critical role in p53 function.

X-ray crystallography is providing key insights into the structure-function significance of *p53 *mutations [36]. An emerging picture is that p53 functions as homotetrameric complexes interacting with the DNA helix, and a multitude of regulatory proteins. DNA-p53 and p53-p53 monomer contacts are important in stabilizing the tetrameric complex. The tetramer is composed of two p53 dimers each straddling the DNA helix. The dimers come together through binding closely spaced DNA decameric half-sites [37,38]. The resulting dimer-dimer is further stabilized by specific p53-p53 interaction sites. G199, which is located in such a key interface termed "patch I", makes critical contacts with residues of the adjacent p53 monomer [39].

To evaluate the effect of the arginine substitution, *in silico *energy minimization within a 10Å sphere surrounding G199R was performed using the Molecular Operating Environment (Figure 4B). Although the analysis anticipates only a subtle conformational change in the interface site, the electrostatic environment is significantly altered. The arginine residue affects interactions with other amino acids much more than the neutral glycine residue originally did. The modeling predicts that the substitution of arginine for glycine at position 199 will neutralize negative charges contributed by nearby inter- and intramonomeric glutamate residues (E171 and E198, respectively). It is therefore likely that G199R would destabilize the tetramer, acting in a dominant negative manner reducing its DNA affinity, and disrupting cooperative interactions between its subunits, and perhaps regulatory proteins. Taken together, the above observations suggest that G199R probably contributed to the molecular events leading to the development of sebaceous carcinoma in this patient.

In summary, the present case emphasizes that young age should not exclude consideration of sebaceous carcinoma. Further studies are needed to determine if sebaceous carcinoma may arise through different sets of environmental factors.

## Consent

Next of kin could not be reached in order to gain consent. There is no reason is suggest that consent would not be given, and all efforts have been made to maintain anonymity.

## Competing interests

The authors declare that they have no competing interests.

## Authors' contributions

DJS took a primary role in writing the manuscript. SAK treated patients and provided the clinical history. FGF performed histological and molecular studies, and participated in the writing. All authors read and approved the final manuscript.
